# α-Klotho Supplementation Mitigates Cumulative Exercise-Induced Fatigue via Coordinated NRF2-Mediated Antioxidant Defense and AKT/GS-Driven Hepatic Glycogen Supercompensation in Mice

**DOI:** 10.3390/ijms27010412

**Published:** 2025-12-30

**Authors:** Lifang Zheng, Yinian Wang, Zirui Xiao, Zhijian Rao, Rengfei Shi

**Affiliations:** 1 Department of Kinesiology, College of Physical Education, Shanghai University, Shanghai 200444, China; 2 Department of Kinesiology, College of Physical Education, Shanghai Normal University, Shanghai 200234, China; 3 Department of Kinesiology, College of Exercise and Health, Shanghai University of Sport, Shanghai 200238, China

**Keywords:** α-klotho, exercise, fatigue, glycogen synthesis, NRF2 pathway

## Abstract

Exercise-induced fatigue involves oxidative stress and metabolic dysregulation. While the anti-aging protein α-Klotho regulates metabolism and oxidative stress, its role in exercise fatigue is unexplored. This study investigated whether α-Klotho supplementation mitigates cumulative exercise-induced fatigue and elucidated the underlying tissue-specific mechanisms. Male C57BL/6J mice were divided into three groups (*n* = 10 per group), the control group, fatigue treated with saline, or α-Klotho (0.2 mg/kg, i.p. daily) group. Fatigue was induced by a 6-day exhaustive swimming protocol (5% body weight load). Tissues were collected 24h post-final exercise. Assessments included daily exhaustion time, grip strength, serum creatine kinase (CK), urea nitrogen (BUN), oxidative stress markers (H_2_O_2_, MDA, SOD, GSH/GSSG), tissue glycogen, and pathway protein expression (Western blot). α-Klotho supplementation prevented exercise-induced weight loss and restored grip strength. While exhaustive exercise markedly increased serum CK and BUN levels, α-Klotho selectively normalized CK without effecting serum BUN. α-Klotho attenuated oxidative damage by reducing hydrogen peroxide levels while enhancing antioxidant capacity, accompanied by activation of the NRF2/HO-1 pathway and further upregulation of PGC-1α. Notably, α-Klotho induced striking hepatic glycogen supercompensation through activation of the AKT/GS signaling pathway and upregulation of GLUT4, whereas muscle glycogen levels remained unchanged. In conclusion, α-Klotho ameliorates cumulative exercise-induced fatigue through dual recovery-phase mechanisms: NRF2/HO-1-mediated antioxidant protection in skeletal muscle and AKT/GS-triggered hepatic glycogen supercompensation, thereby facilitating oxidative stress resolution and enhancing energy reserve restoration.

## 1. Introduction

Aging, metabolic disorders, and oxidative stress are major factors contributing to the decline of physiological function and exercise performance. Among the molecules linked to aging and metabolic regulation, Klotho—also known as α-klotho—has gained increasing attention as a multifunctional anti-aging protein. α-Klotho is predominantly expressed in the kidney and the choroid plexus of the brain, with lower expression in skeletal muscle, adipose tissue, and other organs [1]. It plays a critical role in maintaining renal function, and its deficiency contributes to renal fibrosis and diabetic nephropathy, whereas α-Klotho supplementation alleviates kidney injury [2,3,4,5,6,7]. Beyond the kidney, α-Klotho exerts cardioprotective and myoprotective effects by reducing pathological remodeling and preserving muscle regenerative capacity [8,9,10,11].

Increasing evidence suggests that α-Klotho also serves as a key metabolic regulator [12,13]. Low α-Klotho levels are associated with insulin resistance and hyperglycemia in obesity and type 2 diabetes, while α-Klotho administration improves glucose homeostasis by enhancing insulin secretion, promoting hepatic glycogen synthesis, and facilitating glucose uptake [14,15,16,17]. These effects are thought to involve activation of the phosphoinositide 3-kinase/protein kinase B (PI3K/AKT) pathway, which regulates glucose transport and glycogen metabolism [18,19,20,21,22,23,24,25].

Moreover, α-Klotho provides antioxidant protection through modulation of redox homeostasis. Its overexpression increases superoxide dismutase (SOD) activity and decreases reactive oxygen species (ROS) production [26,27], largely via nuclear factor E2 related factor 2 (NRF2) pathway activation, which induces antioxidant enzymes such as SOD and heme oxygenase-1 (HO-1) [28,29,30,31,32]. Conversely, α-Klotho deficiency downregulates NRF2 and aggravates oxidative stress [8,27,29].

Exercise exerts profound health benefits when performed at appropriate intensity; however, excessive or prolonged high-intensity exercise often induces exercise-related fatigue, driven by metabolic imbalance and oxidative stress [33,34]. Despite extensive studies on α-klotho’s metabolic and antioxidative roles, its potential involvement in exercise-induced fatigue remains largely unexplored, and findings on its regulation by physical activity are inconsistent.

Therefore, this study aimed to investigate whether α-Klotho supplementation attenuates cumulative fatigue induced by short-term high-intensity swimming exercise in mice, and to elucidate the underlying mechanisms related to oxidative stress and glycogen metabolism. Our results suggest that α-Klotho supplementation mitigates fatigue and enhances antioxidant and glycogen-regulating pathways.

## 2. Results

### 2.1. α-Klotho Protects Against Exercise-Induced Fatigue

**Figure 1:**
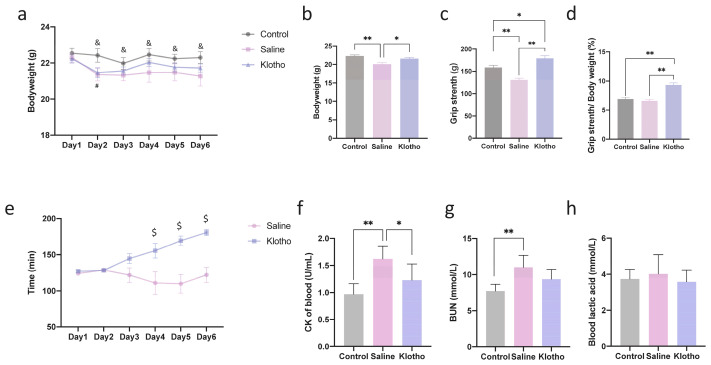
α-Klotho treatment protects against exercise-induced fatigue. (**a**) Changes in mice bodyweight during 6-day exercise training (*n* = 10 per group), (**b**) Mice bodyweight before sacrifice (day 7) (*n* = 10 per group), (**c**) Grip strength of mice (*n* = 10 per group), (**d**) Grip strength normalized by bodyweight, (**e**) Changes of time to exhaustion during exercise training (*n* = 10 per group), changes of blood CK (**f**), BUN (**g**), and lactic acid (**h**) among groups (*n* = 10 per group). Data represented as mean ± SD. * *p* < 0.05, ** *p* < 0.01; α-Klotho vs. Control, # *p* < 0.05; Saline vs. Control, & *p* < 0.05; α-Klotho vs. Saline, $ *p* < 0.05.

After six days of exhaustive swimming exercise, mice exhibited significant weight loss compared with the control group (Figure 1a,b). α-Klotho administration effectively attenuated this weight loss, and the final body weight in the α-klotho-treated group was comparable to controls (*p* = 0.509). Grip strength was markedly reduced in saline-treated exercised mice (*p* = 0.002 vs. control) but was significantly improved by α-Klotho treatment (*p* < 0.0001 vs. saline and *p* = 0.020 vs. control; Figure 1c). Notably, when grip strength was normalized to body weight (Figure 1d), the relative grip strength in the α-klotho-treated group was significantly higher than that in both the control (*p* = 0.0003) and saline-treated (*p* < 0.0001) groups, while no significant difference was observed between the control and saline-treated groups (*p* = 0.776). Time to exhaustion gradually declined over six days in the saline group but was progressively improved by α-Klotho from day 4 onwards (*p* = 0.034, *p* = 0.001, *p* < 0.0001 vs. saline; Figure 1e). Serum creatine kinase (CK) and blood urea nitrogen (BUN) levels, both elevated by exercise (*p* < 0.0001, *p* = 0.002 vs. control), were normalized in the α-klotho-treated group (*p* = 0.109, *p* = 0.065 vs. control; Figure 1f,g). No significant differences in blood lactate levels were observed among groups (*p* > 0.05; Figure 1h). These findings indicate that α-Klotho supplementation alleviates the physical and biochemical manifestations of cumulative exercise-induced fatigue.

### 2.2. α-Klotho Protects Against Exercise-Induced Oxidative Stress in Muscle

**Figure 2:**
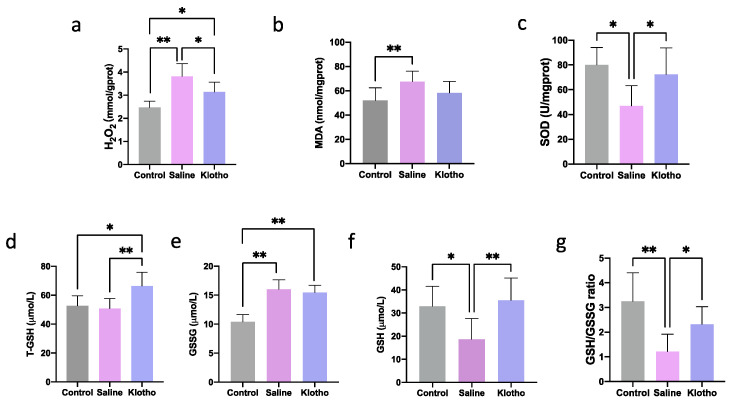
α-Klotho treatment attenuates oxidative stress in muscle. Changes of H_2_O_2_ (**a**), MDA (**b**), SOD (**c**), T-GSH (**d**), GSSG (**e**), GSH (**f**), and GSH/GSSG (**g**) in muscle (*n* = 10 per group). Data represented as mean ± SD. * *p* < 0.05, ** *p* < 0.01.

We next evaluated the impact of exhaustive exercise training on muscle oxidative stress and the potential mitigating effect of α-Klotho supplementation. As shown in Figure 2, compared with the control group, exhaustive exercise significantly increased hydrogen peroxide (H_2_O_2_, Figure 2a, *p* < 0.0001), malondialdehyde (MDA, Figure 2b, *p* = 0.009), and oxidized glutathione (GSSG, Figure 2e, *p* < 0.0001) levels, while it significantly decreased the activity of SOD (Figure 2c, *p* = 0.013), the content of reduced glutathione (GSH, Figure 2f, *p* = 0.035), and the GSH/GSSG ratio (Figure 2g, *p* = 0.001), indicating a state of pronounced oxidative stress. Importantly, treatment with α-Klotho effectively reversed these exercise-induced alterations. Specifically, compared to the Saline group, α-Klotho administration significantly lowered H_2_O_2_ levels (Figure 2a, *p* = 0.014) and markedly elevated SOD activity (Figure 2c, *p* = 0.029), total glutathione (T-GSH, Figure 2d, *p* = 0.003), GSH content (Figure 2f, *p* = 0.005), and the GSH/GSSG ratio (Figure 2g, *p* = 0.040), restoring them to levels close to those observed in the control group. These findings demonstrate that α-Klotho supplementation can partially attenuate exhaustive exercise-induced muscular oxidative stress damage, potentially through enhancing the antioxidant defense system.

### 2.3. α-Klotho Activates NRF2/HO-1 Signaling Pathway in Muscle

**Figure 3:**
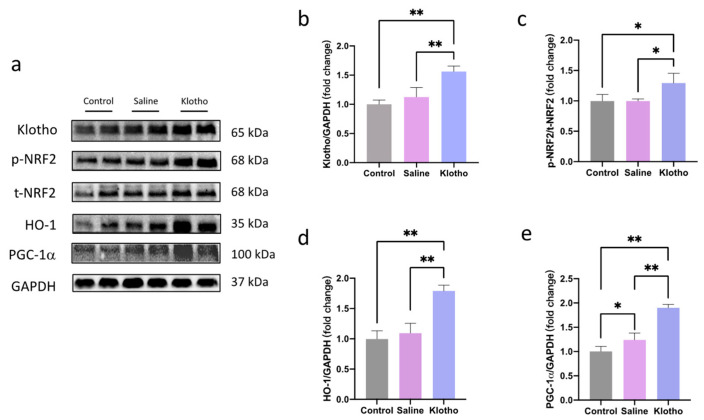
α-Klotho activates NRF2/HO-1 pathway in muscle. (**a**) Representative western blot band. Expression of α-klotho (**b**), p-NRF2/t-NRF2 (**c**), HO-1 (**d**), and PGC-1α (**e**) in muscle (*n* = 6 per group). Data represented as mean ± SD. * *p* < 0.05, ** *p* < 0.01.

Western blot analysis showed that α-Klotho administration increased muscle α-Klotho protein levels (*p* = 0.001 vs. saline; Figure 3a,b). NRF2 phosphorylation (*p* = 0.0126, *p* = 0.0132) and HO-1 (*p* = 0.0001, *p* < 0.0001) expression were significantly enhanced in the α-Klotho group compared with saline or control groups, while no significant differences in NRF2 phosphorylation or HO-1 were observed between control and saline groups (*p* = 0.999, *p* = 0.575; Figure 3c,d). Furthermore, exercise alone increased muscle peroxisome proliferator-activated receptor γ coactivator 1-alpha (PGC-1α) expression (*p* = 0.034 vs. control), and α-Klotho further augmented this effect (*p* < 0.0001 vs. saline; Figure 3e), suggesting α-Klotho promotes mitochondrial and oxidative adaptations through NRF2/HO-1 and PGC-1α pathways.

### 2.4. α-Klotho Increases Glycogen Content in Liver but Not in Muscle

**Figure 4:**
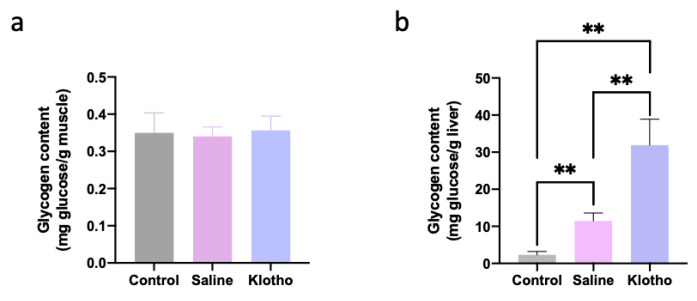
α-Klotho increases liver glycogen content. Glycogen content in muscle (**a**) and the liver (**b**) (*n* = 10 per group). Data represented as mean ± SD. ** *p* < 0.01.

Muscle glycogen content was not significantly altered by exercise or α-Klotho treatment (*p* = 0.891, *p* = 0.956; Figure 4a). In contrast, liver glycogen content increased approximately five-fold in saline-treated exercised mice compared with controls (*p* = 0.007) and was further augmented to approximately 14-fold in α-klotho-treated mice (*p* < 0.0001 vs. both control and saline; Figure 4b), indicating that α-Klotho induces hepatic glycogen supercompensation during recovery.

### 2.5. α-Klotho Promotes Glucose Uptake and Glycogen Synthesis in Liver by Activating AKT Signaling Pathway

**Figure 5:**
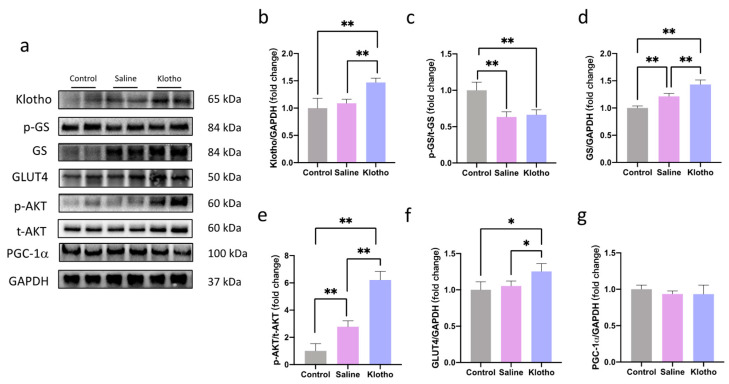
α-Klotho activates the AKT signaling pathway in the liver. (**a**) Representative western blot band. Expression of α-klotho (**b**), p-GS/t-GS (**c**), t-GS (**d**), p-AKT/t-AKT (**e**), GLUT4 (**f**), and PGC-1α (**g**) in the liver (*n* = 6 per group). Data represented as mean ± SD. * *p* < 0.05, ** *p* < 0.01.

In the liver, α-Klotho supplementation increased α-Klotho protein levels (*p* = 0.004 vs. saline; Figure 5a,b) and enhanced phosphorylation of AKT (*p* < 0.0001; *p* < 0.0001) and glycogen synthase (GS) (*p* < 0.0001; *p* = 0.002) compared with both control and saline groups (Figure 5c–e). Glucose transporter 4 (GLUT4) expression was also significantly upregulated by α-Klotho (*p* = 0.013 vs. control, *p* = 0.041 vs. saline; Figure 5f). No significant changes in hepatic PGC-1α were observed among groups (*p* > 0.05).

## 3. Discussion

We explored the effects of the α-Klotho administration on exercise-induced fatigue. Firstly, we found that treatment with α-Klotho after exercise training can reduce exercise fatigue and promote exercise performance. Secondly, α-Klotho attenuates exercise-induced fatigue by, at least partially, activating NRF2/HO-1 pathway to reduce oxidative stress in skeletal muscle. In addition, α-Klotho also protect against exercise-induced fatigue by activating the AKT signaling pathway to increase glucose uptake and glycogen synthesis in the liver.

α-Klotho deficiency caused the decline of muscle strength and endurance performance of mice [35], indicating that the expression level of α-Klotho may affect the fitness of mice. Both human and animal experiments have confirmed that acute exercise can cause an increase in circulating α-Klotho levels Moreover, this exercise-induced effect on α-Klotho appears to be sustainable. Previous studies have consistently reported that athletes have higher serum α-Klotho levels compared with the sedentary population [36,37], and long-term exercise training has been shown to elevate serum α-Klotho levels in both humans and animals [38,39,40,41,42]. These findings collectively suggest that regular, moderate exercise may help maintain α-Klotho at a relatively high baseline level. However, this adaptive response has its limits. Exposure to excessive stress, such as military operational stress and short-term prolonged strenuous exercise training, can lead to a decline of α-Klotho level [43]. Our previous research aligns with this concept, showing that acute exhaustion exercise reduces α-Klotho expression in multiple tissues of mice immediately and at 12 h post-exercise, but it returns to levels comparable to the sedentary control group by 24 h [44]. Therefore, exercise-induced acute fatigue may be associated with a low level of α-klotho, which in turn impairs exercise performance. Consequently, α-Klotho supplementation during intensive exercise training may represent a potential strategy to mitigate fatigue and enhance performance.

This study found that exhaustive exercise for six consecutive days would lead to fatigue in mice, including decreased body weight and grip strength and increased serum CK and BUN. Fatigue disturbs training progress and decreases exercise performance [45]. We found that α-Klotho administration can attenuate the fatigue of mice following exhaustive exercise. In addition, our results also show that exhaustive exercise for six consecutive days does not significantly improve the exercise performance of mice, while the supplement of α-Klotho during exercise can significantly improve the exercise performance of mice. The mechanism of fatigue caused by exhaustive exercise is very complex, which may be related to the imbalance of oxidative stress [34,46]. It is reported in the literature that the increase of free radicals in the body after exhaustive exercise leads to cell damage [47,48]. SOD and GSH are important antioxidant enzymes in the body [49]. Activating SOD and GSH can improve the anti-fatigue ability of the body, thereby improving exercise performance [47,50,51]. In our study, we also observed that six consecutive days of exhaustive exercise increased free radicals in skeletal muscle, and the antioxidant system was inhibited. The treatment of α-Klotho during exercise training can significantly activate the antioxidant system and reduce the free radicals in skeletal muscle. These results indicate that α-Klotho can reduce the oxidative stress of skeletal muscle caused by exhaustive exercise training. Previously, literature reported that α-Klotho has an antioxidant effect in various tissues [21,31,52,53]. The antioxidant effect of α-Klotho may be achieved by activating the NRF2/HO-1 signaling pathway [28,29,30,31]. Our results also showed that the NRF2/HO-1 signaling pathway in the skeletal muscle of mice was significantly activated after α-Klotho administration. Collectively, our results suggest that training supplemented with α-Klotho can activate the NRF2/HO-1 signaling pathway to reduce skeletal muscle oxidative stress.

PGC-1α is essential in mediating exercise adaptation, promoting mitochondrial biosynthesis, muscle fiber type conversion, glucose metabolism, and lipid metabolism adaptation [54,55,56,57]. In the current study, we observed that six consecutive days of exercise training could slightly upregulate (statistically significant) the expression level of PGC-1α in skeletal muscle. In contrast, the supplement of α-Klotho during exercise training can further upregulate the expression of PGC-1α in skeletal muscle. These results suggest that α-Klotho may improve exercise performance by increasing the expression level of PGC-1α in skeletal muscle. However, it is unclear whether α-Klotho directly or indirectly regulates the expression of PGC-1α. Since our results show that α-Klotho cannot upregulate the expression of PGC-1α in the liver, we speculate that α-Klotho may indirectly affect the expression of PGC-1α in skeletal muscle. However, the specific mechanism remains to be further studied.

Glycogen is the primary way for the body to store glucose. Glycogen can be rapidly decomposed to meet the body’s energy needs. During exhaustive exercise, liver glycogen and muscle glycogen in the body will be depleted, which is one of the reasons causing fatigue [58,59,60]. Therefore, increasing glycogen levels can effectively attenuate fatigue [59,61]. In addition, increasing glycogen contents before exercise is an important strategy to improve exercise performance and prevent exercise fatigue [58,62,63]. α-Klotho was reported to play a role in regulating the metabolism [12,13]. In vivo and in vitro studies have demonstrated that α-Klotho can promote glucose uptake and glycogen synthesis in the liver [14]. We found that six days of exhaustive exercise training did not increase skeletal muscle glycogen content, regardless of α-Klotho supplementation. However, six days of strenuous exercise training can significantly increase the liver glycogen content, and the treatment of α-Klotho can further increase the liver glycogen content in trained mice. In addition, our results also showed that α-Klotho administration could up-regulate the expression of GLUT4 in the liver of trained mice. However, exhaustive exercise did not significantly affect the expression of GLUT4 in the liver. These results indicate that α-Klotho can increase glucose uptake and glycogen synthesis in the liver. Little is known about the effects of α-Klotho on glucose metabolism. Previously, we found that α-Klotho can regulate food intake and glucose metabolism by activating AKT signaling in the brain [64]. AKT can promote glucose uptake by increasing the expression of GLUTs and translocation to the cell membrane and regulating the activity of downstream glycogen synthase to promote glycogen synthesis [18,19,20]. The results of this study demonstrate that α-Klotho administration during training does activate the AKT pathway and increases GS activity. Collectively, we found that α-Klotho administration can activate the AKT signaling pathway, up-regulate GLUT4 and GS, and promote glycogen synthesis in the liver of trained mice.

There are several limitations to this study. First, we did not examine whether acute α-Klotho administration alone could enhance endurance exercise performance in mice. Although previous studies have reported that skeletal muscle-specific overexpression of α-Klotho did not significantly improve grip strength, endurance capacity was not assessed in those studies. Given the demonstrated antioxidant and glycogen-promoting effects of α-klotho, it is plausible that administering α-Klotho shortly before exercise may enhance endurance performance, which warrants further investigation. Second, we did not evaluate the potential adverse effects of long-term exogenous α-Klotho administration. Previous reports indicate that sustained α-Klotho overexpression may induce insulin resistance, raising the possibility that chronic α-Klotho treatment could disrupt metabolic homeostasis. Therefore, studies examining the metabolic safety of prolonged α-Klotho supplementation are needed. Third, we did not assess the impact of α-Klotho on exercise-induced skeletal muscle inflammation and injury. Since prolonged strenuous exercise is known to cause muscle inflammation and damage, which contribute to fatigue, and given α-klotho’s reported anti-inflammatory and pro-regenerative properties, it would be valuable to determine whether α-Klotho mitigates these pathological processes.

## 4. Materials and Methods

### 4.1. Animals and Experiment Design

Male C57/BL6J mice (seven weeks old) were obtained from Shanghai Model Organisms (Shanghai, China) and maintained under standard laboratory conditions (21 ± 1 °C, 30–40% humidity, 12-h light/dark cycle) in accordance with the National Institutes of Health guidelines for laboratory animal care. The mice were fed a standard laboratory chow diet (energy composition: 75% carbohydrate, 20% protein, 5% fat) ad libitum throughout the entire study period, including before, during, and after the training protocol. The experimental procedures were approved by Institutional Animal Care and Use Committee of Shanghai University of Sport.

Following a seven day acclimatization period, the mice were randomly divided into three experimental groups (10 mice per group a sedentary control group (control), an exhaustive exercise group receiving saline (Saline), and an exhaustive exercise group treated with α-Klotho protein. The latter two groups underwent a daily exhaustive swimming exercises for six consecutive days, with a load equivalent to 5% of their body weight attached to the tail. Prior to the formal intervention, all animals were acclimated to the experimental swimming pool (30 °C, water depth: 30 cm; radius 120 cm) for three days (before the start of the experimental exercise), with swimming duration gradually increased from 10 to 20 min without load, followed by 10–20 min with a 5% bodyweight load. Exhaustion was defined as the inability to return to the water surface for more than 10 s. Saline or recombinant α-Klotho protein (0.02 mg/kg) was administered via intraperitoneal injection 1 h after the exhaustive swimming exercise. This dosage was selected based on our previous study in an obese mouse model, where the same dose (administered every other day) was effective and showed no observable side effects [12]. The administration regimen was adjusted to a daily injection in the present study to maintain a more consistent drug concentration during the acute exercise recovery period. The post-exercise timing was chosen to specifically investigate the therapeutic effect of α-Klotho on recovery from fatigue and exercise-induced oxidative stress. The recombinant α-Klotho protein was obtained from R&D (Cat#:1819-KL-050). It was reconstituted in phosphate-buffered saline (PBS) at a concentration of 0.1 mg/mL. Body weight and exhaustive swimming time were recorded daily. Grip strength of the forelimb was assessed 24 h after the final exercise bout. Briefly, the grip strength of mice was measured using a commercial grip strength meter (YLS-13A, Jinan Yiyan Technology Development Co., Ltd., Jinan, Shandong, China). Each mouse was gently placed on the apparatus to grasp the metal grid with its forepaws, and then pulled backward steadily by the tail until it lost its grip. The test was repeated three times for each mouse with a 1-min rest interval, and the average value of the three measurements was calculated and recorded by the instrument automatically. Blood samples were collected via retro-orbital bleeding under deep anesthesia induced by isoflurane inhalation. The blood was placed in standard 1.5 mL centrifuge tubes and allowed to clot at room temperature for 30 min. Subsequently, the samples were centrifuged at 2500× *g* for 15 min at 4 °C to obtain serum. Animals were euthanized by cervical dislocation, and liver and gastrocnemius muscle tissues were rapidly frozen in liquid nitrogen for subsequent analysis.

### 4.2. Biochemical and Physiological Measurements

Serum levels of CK, BUN, and lactic acid were measured using commercial assay kits (Nanjing Jiancheng Bioengineering Institute, Nanjing, China, Cat# A032-1-1, C013-2-1, A019-1-1). The detailed procedures for each assay are described below.

Lactic acid: The concentration of lactic acid in whole blood was determined based on the principle that lactate dehydrogenase (LDH) catalyzes the oxidation of lactate to pyruvate, concomitantly reducing NAD+ to NADH. The generated NADH then transfers hydrogen via phenazine methosulfate (PMS) to reduce nitroblue tetrazolium (NBT), producing a purple formazan dye. The absorbance of this dye at 530 nm is proportional to the lactic acid concentration. Briefly, fresh whole blood samples (0.1 mL) were immediately deproteinized by adding 0.6 mL of protein precipitant reagent. The mixture was vortexed and centrifuged at 3500–4000 rpm for 10 min. The resulting supernatant was used for the assay. The reaction mixture, containing 0.02 mL of supernatant, 1 mL of enzyme working solution (prepared freshly by mixing enzyme stock solution and enzyme diluent at a 1:100 ratio), and 0.2 mL of color developer, was incubated at 37 °C for exactly 10 min. The reaction was terminated by adding 2 mL of stop solution. Absorbance was measured at 530 nm against a blank. The lactic acid concentration (mmol/L) was calculated using the formula: (A_sample − A_blank)/(A_standard − A_blank) × C_standard (3 mmol/L).

BUN: The BUN level was quantified using the urease method. Urea is hydrolyzed by urease in the buffer enzyme solution to produce ammonium ions. In an alkaline medium, these ions react with phenol and hypochlorite to form a blue indophenol complex, the absorbance of which at 640 nm is directly proportional to the urea concentration. For the assay, 0.02 mL of plasma sample was mixed with 0.25 mL of buffer enzyme solution (prepared freshly before use) and incubated at 37 °C for 10 min. Then, 1 mL of phenol color reagent and 1 mL of alkaline sodium hypochlorite were added sequentially. After thorough mixing, the solution was incubated at 37 °C for another 10 min. The absorbance was measured at 640 nm. The BUN concentration (mmol/L) was calculated as: (A_sample − A_blank)/(A_standard − A_blank) × C_standard (10 mmol/L). If necessary, samples were diluted with physiological saline.

CK: The activity of CK-MB isoenzyme was determined by an immunoinhibition method. An anti-M antibody was used to inhibit the M subunit of CK-MM and CK-MB. The remaining activity of the B subunit (from CK-BB and CK-MB) was then measured kinetically. Since CK-BB is minimally present in serum, the measured activity primarily reflects CK-MB, which is multiplied by 2 to estimate the total CK-MB activity. The reaction is coupled to the reduction of NADP+ to NADPH, monitored by the increase in absorbance at 340 nm. For manual spectrophotometric assay, 40 μL of serum sample was mixed with 800 μL of reagent R1 (containing substrates, cofactors, enzymes, and the anti-M antibody) and 200 μL of reagent R2 (containing phosphocreatine). The mixture was incubated at 37 °C for 2 min, and the initial absorbance (A1) was recorded. After a further 3-min incubation, the final absorbance (A2) was measured. The change in absorbance per minute (ΔA/min) was calculated. CK-MB activity (U/L) was determined using the formula: ΔA/min × (Total reaction volume/Sample volume) × (1000/6.22) × 2 = ΔA/min × 8360. All assays were performed in duplicate.

Oxidative stress markers, including MDA and H_2_O_2_ content, were determined using specific reagent kits (Cat# A003-1-2 and A064-1-1). SOD activity was evaluated with a corresponding kit (Cat# A001-3-2). T-GSH and GSSG levels were measured (Cat# A061-1-2), and GSH concentration was calculated as T-GSH minus 2× GSSG. The detailed procedures for each assay are described below.

MDA: Lipid peroxidation was assessed by measuring malondialdehyde (MDA) levels using the thiobarbituric acid reactive substances (TBARS) method. Briefly, samples were incubated with thiobarbituric acid under acidic conditions at 95 °C for 40 min. The resulting pink chromogen was extracted and measured spectrophotometrically at 532 nm. MDA concentrations were calculated using a standard curve generated with tetraethoxypropane and expressed as nmol/mg protein.

H_2_O_2_ levels: H_2_O_2_ content was determined using a colorimetric method (Kit No. A064-1-1) based on the peroxide-mediated oxidation of ferrous ions followed by complex formation with a dye reagent. Absorbance was measured at 405 nm, and H_2_O_2_ concentrations were calculated against a standard curve and normalized to protein content (mmol/g protein).

SOD activity: SOD activity was evaluated using the WST-1 method (Kit No. A001-3-2), which measures the inhibition of superoxide anion-mediated reduction of WST-1 to formazan. The assay was conducted in 96-well microplates with incubation at 37 °C for 20 min, and absorbance was read at 450 nm. One unit of SOD activity was defined as the amount of enzyme required to inhibit the reduction reaction by 50% under assay conditions. Results were expressed as U/mg protein.

T-GSH and GSSG levels were determined using an enzymatic recycling assay (Kit No. A061-1-2). The method utilizes glutathione reductase and DTNB, where the rate of TNB formation is proportional to total glutathione concentration. For GSSG measurement, reduced glutathione was masked prior to analysis. Reduced glutathione (GSH) content was calculated using the formula: GSH = T-GSH − 2 × GSSG. Concentrations were normalized to protein content and expressed as μmol/g protein.

All the aforementioned assays were performed one week after the tissue samples were snap-frozen and stored at −80 °C.

### 4.3. Tissue Glycogen Determination

Hepatic and muscular glycogen levels were quantified using a glycogen assay kit (Nanjing Jiancheng Bioengineering Institute, Nanjing, China, Cat# A043-1-1), following the manufacturer’s instructions. Briefly, the assay is based on the anthrone-sulfuric acid method, where glycogen is hydrolyzed and dehydrated to form furfural derivatives that react with anthrone to generate a blue-colored complex, measurable at 620 nm. Tissue samples (≤100 mg) were hydrolyzed in alkaline solution (tissue weight: hydrolysis solution volume = 1:3) by boiling for 20 min. The hydrolysates were then diluted with distilled water to prepare 1% (liver) or 5% (muscle) glycogen test solutions. After mixing with the anthrone-sulfuric acid reagent, the samples were heated in a boiling water bath for 5 min, cooled, and the absorbance was measured. Glycogen content (mg/g tissue) was calculated as (OD_sample/OD_standard) × (0.01 mg) × dilution factor × (1/1.11).

### 4.4. Western Blot Analysis

Western blotting was conducted as previously outlined [44]. Total protein was extracted from muscle tissues using RIPA lysis buffer supplemented with protease and phosphatase inhibitors. The protein concentration was determined using a bicinchoninic acid (BCA) assay kit (Beyotime, Shanghai, China, cat# P0006), following the manufacturer’s instructions. Equal amounts of protein (40 µg per lane) were separated by SDS-PAGE on 10% or 12% gels and subsequently transferred onto PVDF membranes (Millipore, Hoeilaart, Belgium).

After blocking with 5% non-fat milk, the membranes were incubated at 4 °C overnight with the following primary antibodies (all diluted 1:1000): α-Klotho (R&D Systems, Minneapolis, MN, USA, Cat# AF1819), NRF2 (Proteintech, Wuhan, Hubei, China, Cat # 16396-1-AP), p-NRF2 (Abways, Shanghai, China, Cat # CY6573), HO-1 (GenTex, Irvine, CA, USA, Cat # GTX13248), PGC-1α (Proteintech, Wuhan, Hubei, China, Cat # 66369-1-Ig), p-GS (Proteintech, Wuhan, Hubei, China, Cat # 28855-1-AP), GS (Proteintech, Wuhan, Hubei, China, Cat # 10566-1-AP), GLUT4 (Proteintech, Wuhan, Hubei, China, Cat # 66846-1-Ig), p-AKT (CST, Danvers, MA, USA, Cat # 4060S), t-AKT (CST, Danvers, MA, USA, Cat # 9272S), and GAPDH (CST, Danvers, MA, USA, Cat # 2118S). Following washes, the membranes were incubated with an HRP-conjugated goat anti-rabbit (or anti-mouse, as appropriate) secondary antibody (diluted 1:5000) for 1 h at room temperature. Protein bands were visualized using an enhanced chemiluminescence (ECL) kit (Thermo Fisher Scientific, Waltham, MA, USA) and imaged with a chemiluminescence imaging system (Bio-Rad Imaging System, Hercules, CA, USA). The band intensities were quantified using ImageJ software (version 1.5.4; National Institutes of Health, Bethesda, MD, USA).

### 4.5. Statistical Analysis

All quantitative data are expressed as mean ± standard deviation (SD). The normality of the data distribution was assessed using the Shapiro-Wilk test, and the homogeneity of variances was verified using Levene’s test. For data that satisfied both normality and homogeneity of variances, one-way analysis of variance (ANOVA) was performed to determine the overall differences among groups. When the ANOVA result was significant, Tukey’s honestly significant difference (HSD) post-hoc test was applied for multiple comparisons between groups to control the family-wise error rate. A *p* values less then 0.05 was considered statistically significant.

## 5. Conclusions

In summary, this study demonstrates that α-Klotho supplementation effectively ameliorates cumulative exercise-induced fatigue in mice by engaging dual recovery-phase mechanisms. α-Klotho reduced skeletal muscle oxidative damage by activating the NRF2/HO-1 signaling pathway and enhancing antioxidant defenses, while promoting hepatic glycogen supercompensation through AKT/GS signaling and GLUT4-mediated glucose uptake. Together, these findings reveal that α-Klotho improves both redox homeostasis and energy reserve restoration following strenuous exercise, highlighting its potential as a therapeutic strategy to enhance exercise adaptation and performance.

## Data Availability

The original contributions presented in this study are included in the article. Further inquiries can be directed to the corresponding authors.

## Abbreviations

The following abbreviations are used in this manuscript:

CK	Creatine Kinase
BUN	Blood Urea Nitrogen
H_2_O_2_	Hydrogen Peroxide
MDA	Malondialdehyde
GSSG	Oxidized Glutathione
SOD	Superoxide Dismutase
GSH	Glutathione (Reduced)
NRF2	Nuclear factor erythroid 2-related factor 2
HO-1	Heme Oxygenase-1
PGC-1α	Peroxisome proliferator-activated receptor gamma coactivator 1-alpha
AKT	Protein Kinase B
GS	Glycogen Synthase
GLUT4	Glucose Transporter type 4
